# *GATA5* CpG island hypermethylation is an independent predictor for poor clinical outcome in renal cell carcinoma

**DOI:** 10.3892/or.2014.3030

**Published:** 2014-02-18

**Authors:** INGA PETERS, KAI GEBAUER, NATALIA DUBROWINSKAJA, FARANAZ ATSCHEKZEI, MARIO W. KRAMER, JOERG HENNENLOTTER, HOSSEIN TEZVAL, MAHMOUD ABBAS, RALPH SCHERER, AXEL S. MERSEBURGER, ARNULF STENZL, MARKUS A. KUCZYK, JUERGEN SERTH

**Affiliations:** 1 Department of Urology and Urologic Oncology, Hannover Medical School, D-30625 Hannover, Germany; 2 Department of Pathology, Hannover Medical School, D-30625 Hannover, Germany; 3 Department of Biometry, Hannover Medical School, D-30625 Hannover, Germany; 4 Department of Urology, Eberhard-Karls University, D-72076 Tuebingen, Germany

**Keywords:** *GATA3*, *GATA5*, renal cell cancer, DNA hypermethylation, survival, prognosis

## Abstract

Transcriptional inactivation and CpG island (CGI) methylation of GATA transcription factor family members *GATA3* and *GATA5* have been reported for a few types of human cancer. Whether high-density CGI methylation of *GATA3* or *GATA5* is associated with the clinical course of patients with renal cell cancer (RCC) has not been clarified. Quantitative methylation-specific PCR assays were carried out to analyze 25 tumor cell lines including 6 RCC lines and 119 RCC and 87 adjacent normal tissues for the presence of densely methylated sequences. Methylation values were statistically compared with clinicopathological and recurrence-free survival (RFS) data for patients. Comparison of *GATA3* and *GATA5* methylation in different tumor cell lines revealed a marker-specific methylation characteristic with high and frequent signals for both methylation marks in RCC lines. *GATA3* and *GATA5* CGI relative methylation levels were found to be strongly associated with the state of metastasis (P=0.003 and P<0.001, respectively) and advanced disease (P=0.024 and P<0.001, respectively). Moreover, an independent decrease in RFS in Cox proportional hazard analysis was found for tumors exhibiting high *GATA5* methylation (P<0.001, hazard ratio, 19.3; 95% confidence interval, 4.58–81.6). Epigenetic alterations in GATA family members may be associated with aggressive tumor phenotypes in RCC, and in the case of *GATA5*, may serve as a new independent molecular marker for aggressiveness and disease progression.

## Introduction

Renal cell carcinoma (RCC) is the tenth most common cancer in men worldwide (1) and the third most common genitourinary tumor. The use of targeted therapies has improved treatment of metastatic RCC, but survival remains significantly decreased in late-stage or metastatic RCC patients (2).

The molecular carcinogenesis of clear cell renal cell carcinoma (ccRCC) includes *von Hippel-Lindau* gene alterations as gatekeeper mutations that are followed by additional genetic changes for full development of the cancer (3). In view of the epigenetic progenitor cancer model, such mutations may be substituted by epigenetic alterations that cause gene silencing and thus contribute to the accumulation of epigenetic and genetic alterations, as has been found for several human malignancies (4). Indeed, a considerable number of loci undergoing DNA methylation have been identified in ccRCC at a high frequency. For example, the secreted frizzled-related protein (*SFRP1*) and RAS-associated domain family 1 CpG island (CGI) hypermethylation have been found in 34–68% and 28–76% of RCCs, respectively (5–7). Hypermethylation of the *SCUBE3* gene is associated with clinicopathological para- meters and poorer survival (8). A genome-wide CGI methylation analysis by Ricketts *et al* (9) showed that CGI hypermethylation of several genes (including *SLC34A2* in 63%, *OVOL1* in 40%, *DLEC* in 20%, *TMPRSS2* in 26%, *SST* in 31% and *BMP4* in 35% of RCC) is associated with transcriptional silencing, reactivation after demethylation in RCC cell lines and downregulation of expression in RCC.

Recently, we identified *GATA5*, a member of the GATA transcription factor family (GATA1 to GATA6), as a new target for CGI hypermethylation in RCC, also demonstrating a statistical association with disease progression and decreased survival. However, since combined bisulfite restriction analysis detection was applied for methylation detection, only site-specific average methylation could be assessed (10). Heterogeneous methylation as determined in the CGI of *GREM1* in RCC (11) may lead to varying statistical associations with clinicopathological parameters; thus, our previous findings of *GATA5* CGI methylation as a potential prognosticator for RCC would be strengthened if another *GATA5* methylation locus could be identified to demonstrate association with an unfavorable prognosis. Detecting highly methylated sequences located in a different subregion of the *GATA5* CGI would provide further evidence for a crucial role of *GATA5* in RCC progression.

In addition, comparing expression and methylation data from public databases (12), we noted that *GATA3*, as a member of the GATA transcription factor family, might also represent a potential target for CGI hypermethylation. The GATA1, GATA2 and GATA3 members of the GATA transcription factor family are functionally involved in cellular lineage determination (13) while the GATA4, GATA5 and GATA6 are mainly involved in epithelial differentiation and are suggested to play a critical role in tumorigenesis of cancer with endo- or mesodermal origins (13). Furthermore, both mechanisms exhibit extensive changes in neoplastic development in different cancer types (14) and loss of GATA3 expression in breast cancer patients has been significantly associated with poor clinical outcome and advanced tumor disease (15). Comparing normal and tumor renal tissues, decreased GATA3 protein and mRNA expression levels have already been observed, supporting the hypothesis that *GATA3* may be epigenetically silenced in RCC (16).

To clarify the relevance of *GATA3* and *GATA5* methylation in RCC, we measured CGI methylation of both genes in normal human primary tubule epithelial cells and in renal tumor cell lines, as well as in renal cancer tissues and a subset of paired adjacent normal tissues, using quantitative methylation-specific PCR (qMSP). We found that higher methylation is more likely to be found in tumors of patients with advanced and metastatic disease and in case of *GATA5* is also associated with poorer survival of RCC patients.

## Materials and methods

### Tissue specimens

Cross-sectional analyses were conducted on 119 RCC samples and 87 samples from paired histologically normal-appearing tissues, i.e., adjacent normal renal tissue. Tissue samples were collected from patients who had undergone radical or nephron-sparing nephrectomy and stored as previously described (17). TNM classification of all tissues was evaluated according to the Union for International Cancer Control 2010 classification, and grading was assessed as previously described (18,19). Localized RCC was defined as pT ≤2, lymph node (N) and metastasis (M) negative (N0 and M0), and a grading (G) of 1 and 1–2. Advanced tumors were classified as p ≥T3 and/or lymph node positive (N+), positive for distant metastasis (M+) or G2–3 and G3. Time to disease recurrence was designated as the point at which patients had either a local recurrence or a synchronous/metachronous metastasis as detected by computerized tomography scan. The local ethics committee approved sample collection, and informed consent was obtained from each patient. Clinical and histopathological parameters of tissues are summarized in Table I. Purchase, culturing, storage and identity control of cell lines and primary cells were carried out as previously described (17).

### Isolation of DNA and bisulfite conversion

DNA was extracted from frozen tissue sections using a standard phenol/chloroform extraction method. Bisulfite conversions and histopathological examination of control sections were conducted as previously reported (20).

### Quantitative methylation-specific real-time PCR analysis of GATA3 and GATA5 CGI methylation

**Figure 1:**
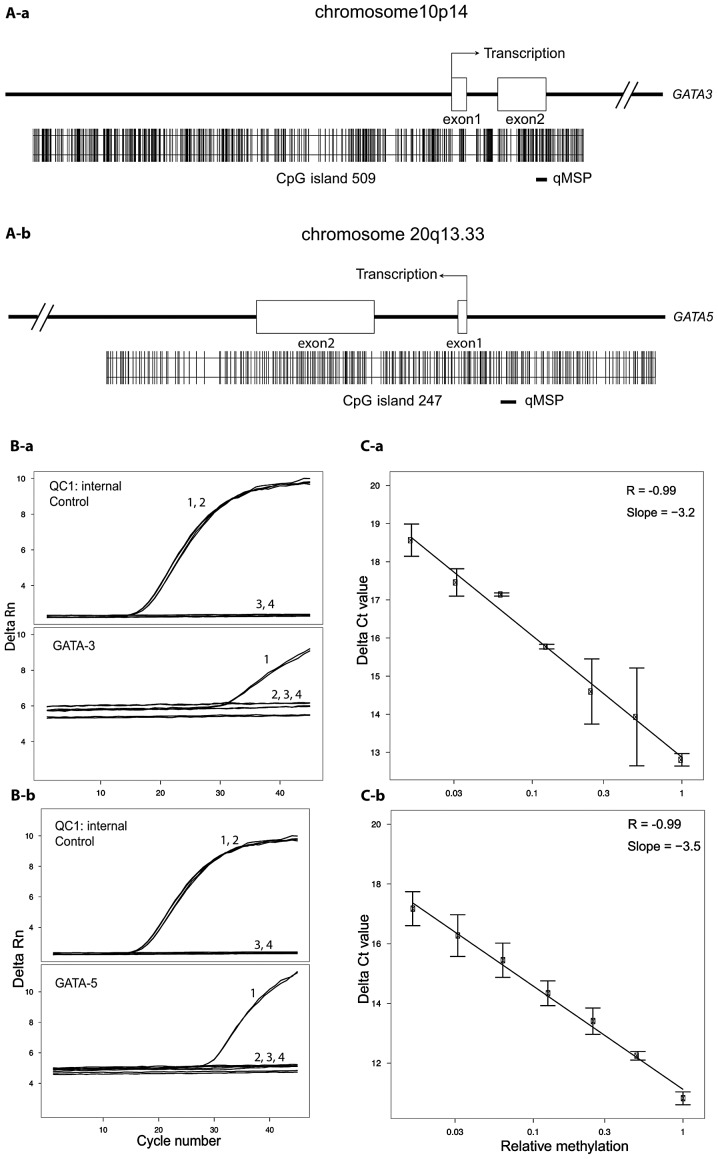
Description of investigated CpG islands of *GATA3* and *GATA5* and assay controls. (A-a) Structure of the *GATA3* CGI locus and location of the qMSP assay relative to the transcription start site. *GATA3* is located on chromosome 10p14. CpG sites are illustrated with vertical lines within the CpG island. Information refers to UCSC Genome Browser database and GenBank (12,22). (A-b) *GATA5* structure and location of the qMSP assay relative to the transcription start site. Vertical lines represent CpG sites within the CpG island. Base positions refer to the GRCh37/hg19 annotation in the UCSC Genome Browser and GenBank (12,22). (B) Primary data of quantitative methylation-specific and control PCR measurements in methylated control DNA (1); unmethylated control DNA (2); unconverted DNA (3); and a blank control (4) for *GATA3* (a) and *GATA5* (b) analysis. (C) Normalized *GATA3* (a) and *GATA5* (b) assay threshold values (Ct) for a 2-fold dilution series of the methylated control in non-methylated control DNA for determination of assay linearity and efficiency.

Methylation analyses of bisulfite-treated genomic DNA for CGI methylation of *GATA3* and *GATA5* was performed by quantitative real-time fluorimetric 5′ exonuclease methylation-specific PCR assays. Methylation analysis was carried out as described elsewhere (21). The qMSP-specific primers 5′-TGTATCGGGACGGA ATCGTT-3′ (forward) and 5′-ACGCGCGCTCTAACCCTT-3′ (reverse) as well as the Taqman^®^ probe 5′-FAM-AAATAT AACCGCGACTCCTACCAATTCATTCG-BHQ-3′ were designed using Beacon Designer™ software (Premier Biosoft, Palo Alto CA, USA). Intra-CGI location of both qMSP assays, designed within an area of high GC percentage, is shown in Fig. 1A-a (*GATA3*) and in Fig. 1A-b (*GATA5*). Table II shows the base positions of investigated CpG sites in the corresponding CGI referenced in the USCS Genome Browser (12,22). Real-time PCR was carried out in duplicate using a FasTrans automatic Liquid Handling System (Analytik Jena, Jena, Germany) and an ABI 7900HT (Life Technologies, Foster City, CA, USA) in 384-well plates as previously reported (17). An experimenter who was blinded to type, order and clinicopathological status of samples carried out measurements.

### Calculation of relative methylation indices and statistical analysis

Calculation of the relative degree of methylation was based on the method of Weisenberger *et al,* recently described in detail (21,23). Statistical analyses and definition of the cut-off value for dichotomization used in survival analysis were also carried out as previously described (17).

For univariate statistical analyses, all groups were dichotomized according to their clinicopathological parameters, i.e., localized (Loc.) vs. advanced (Adv.) disease, metastasis negative (M0) vs. positive (M+), lymph node metastasis-negative vs. lymph node metastasis-positive (N0/N+), and low-grade (G1, G1–2) vs. high-grade (G2–3, G3) tumors.

## Results

### Measurement of technical controls and analysis of GATA3 and GATA5 CGI methylation in human normal cells and cancer cell lines

The specificity of the *GATA3* and *GATA5* qMSP analyses was evaluated by duplicate measurements of converted methylated (M), converted non-methylated (U) and non-converted DNA control samples. For U and non-converted DNA samples, we exclusively measured Ct values of 45 (undetermined) whereas the M sample demonstrated Ct values of ~32 for *GATA3* (Fig. 1B-a) and Ct values of ~29 for *GATA5* (Fig. 1B-b). None of the control or CGI-specific qMSP assays gave signals for non-converted DNA, thus demonstrating that only methylated and converted DNA was detected. PCR efficiency and linearity of the methylation detection assays were assessed using a log dilution series of the M control within the U control DNA and adjusting for constant total converted DNA amount in PCR reactions. Linear regression analyses demonstrated a slope of ΔCt = −3.3 per 10-fold dilution and a coefficient of correlation of r=−0.99 for both genes (P=0.001), indicating linearity of the assays (Fig. 1C-a and 1C-b).

**Figure 2:**
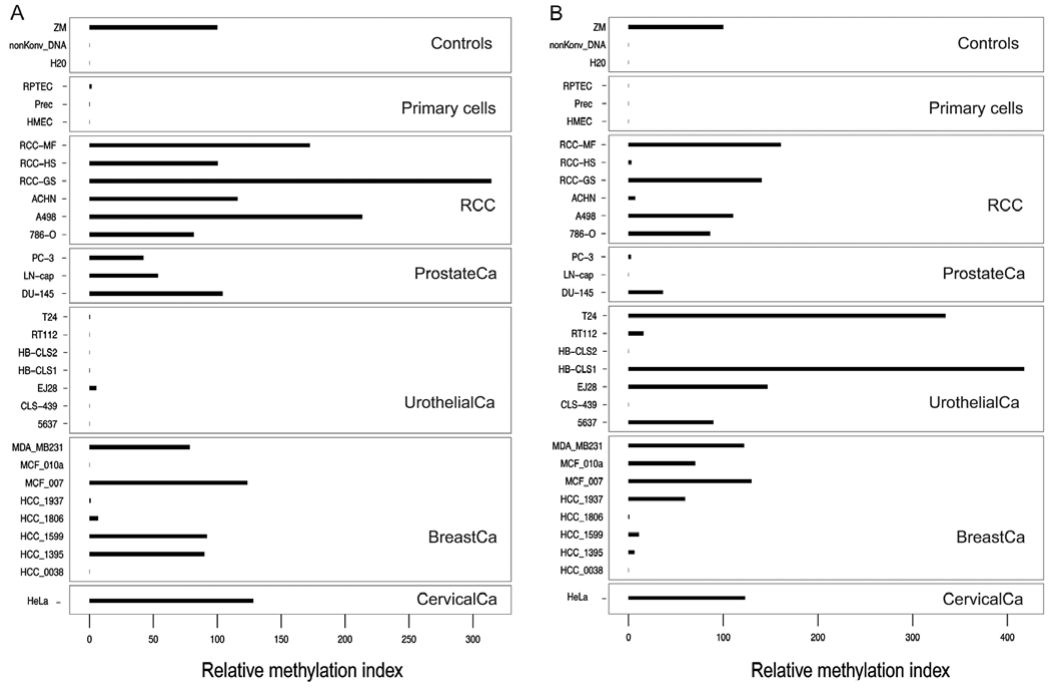
Measurement of relative methylation in different cancer cell lines. Levels of relative methylation values in cancer cell lines and normal primary cells for (A) *GATA3* and (B) *GATA5*.

We assessed whether the *GATA3* and *GATA5* qMSP assays are capable of methylation detection in normal human primary tubule epithelial cells and in cancer cell lines, each respectively used as a proxy for normal tissues and localized and metastatic human cancers of other origin (kidney, prostate, bladder, breast and cervical cancer cell lines), which in part have already been reported to demonstrate tumor specific hypermethylation. Methylation for *GATA3* was found in 5/8 (63%) breast cancer cell lines, as expected from previous reports describing *GATA3* methylation in breast cancer tissue samples. Notably, all 6 renal cancer cell lines showed high relative methylation indices while normal primary cells from kidney (RPTEC), prostate cancer, and mammary tissues demonstrated low or undetectable methylation (Fig. 2A). Similarly, *GATA5* CGI methylation was not detectable or was low in normal primary cells but demonstrated higher relative methylation indices only for 4/6 renal cancer cell lines (Fig. 2B).

### GATA3 and GATA5 CGI is hypermethylated in RCC

**Figure 3:**
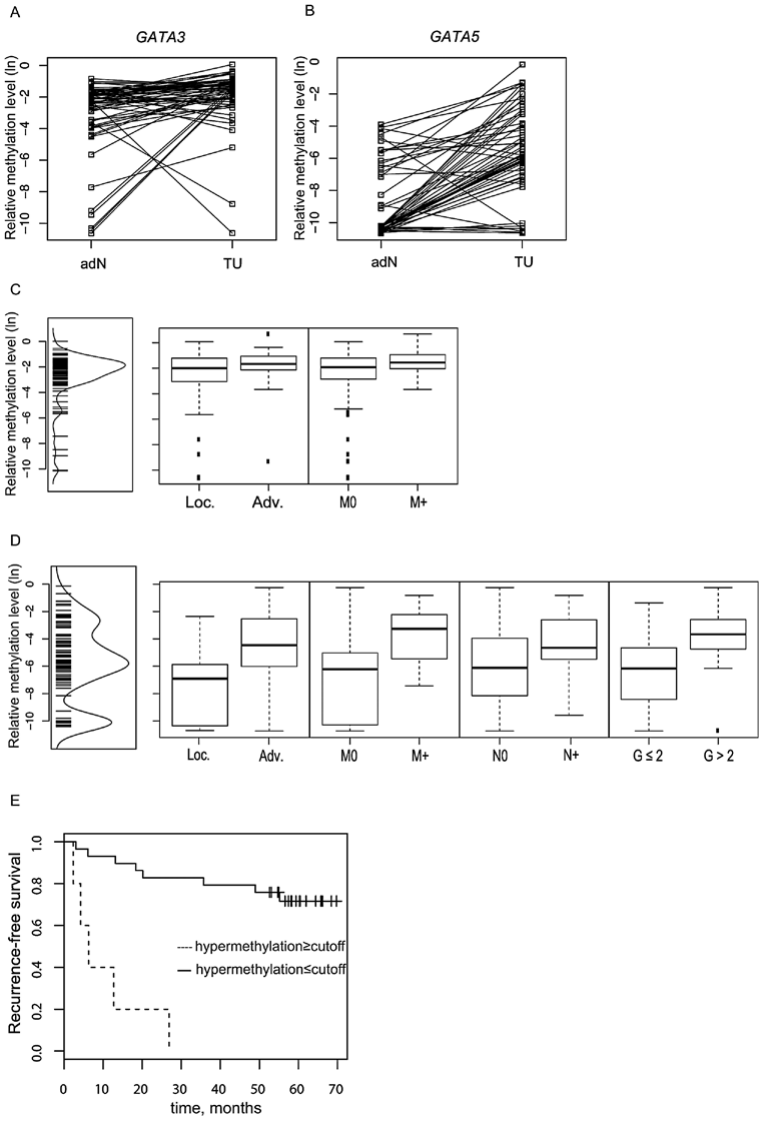
Associations of DNA methylation of *GATA3* and *GATA5* with clinicopathology and recurrence-free survival. Comparison of the natural logarithms of relative methylation values for (A) *GATA3* and (B) *GATA5* in adjacent normal (adN) and tumor (TU) tissues of a ccRCC patient cohort (P=0.001, P<0.001). (C) Box plot illustration of *GATA3* CGI methylation. *GATA3* methylation was significantly increased in advanced (Adv.) and metastasized (M+) renal cell cancer patients compared to localized (Loc.) and non-metastasized (M0) disease (P=0.024 and P=0.003, respectively). Distribution of relative methylation values is illustrated in the Kernel distribution graph. (D) Distribution of *GATA5* methylation values in all RCCs in association with clinicopathological parameters: Loc. and Adv. (P<0.001), M1 and M0 (P<0.001), lymph node status (N0/N+; P=0.03), and high-risk (G >2) and low-risk (G ≤ 2) grade (P=0.003). Distribution of relative methylation values is illustrated in the Kernel distribution graph. (E) Kaplan-Meier plot showing the relative recurrence-free survival of clear cell renal cancer patients with *GATA5* hypermethylation. Results were dichotomized by a cut-off of −2.447. The dashed line indicates the patients with relative methylation value higher than the cut-off of −2.447, demonstrating a significantly decreased recurrence-free survival.

Comparison of *GATA3* and *GATA5* methylation in matched tumor (TU) and adjacent normal (adN) tissues demonstrated tumor-specific hypermethylation (Fig. 3A and B). Using the paired t-test for statistical analysis (Table III), we found significant differences for *GATA3* methylation in the RCC tissue groups (P=0.006) as well as in the ccRCC subset (P=0.001). The corresponding analysis of *GATA5* methylation also demonstrated higher methylation in tumor tissues both for the RCC group (P<0.001) and the ccRCC subset (P<0.001).

### Analysis of GATA3 and GATA5 CGI methylation and association with clinicopathological parameters

Univariate logistic regression analysis (Table III) of dichotomized groups revealed a statistically significant association between methylation of *GATA3* and *GATA5* CGI with advanced and metastasized RCC disease. Mean methylation for both CGIs (*GATA3* and *GATA5*) was significantly higher in advanced vs. localized (P=0.024 and P<0.001, respectively) and in metastasis-negative (M0) vs. metastasis-positive (M+) tumors (P=0.003 and P<0.001, respectively; Fig. 3C and D) of the RCC tissue group. In addition, *GATA5* showed a significantly higher CGI methylation status in the high-grade tumor and positive lymph node metastasis (N+) groups compared to low-grade tumor tissues (P=0.003) or negative lymph node status (P=0.03; Fig. 3D). Comparison of CGI methylation of *GATA3* and *GATA5* in ccRCC and papillary renal cell carcinoma showed significant statistical differences for the mean *GATA3* (P=0.006) and *GATA5* (P=0.015) relative methylation indices observed in both histological entities (Table III).

### GATA5 CGI methylation is independently associated with decreased recurrence-free survival

Univariate Kaplan-Meier and bivariate Cox proportional hazard analysis were conducted to elucidate a possible relationship between *GATA3* and *GATA5* CGI methylation and recurrence-free survival (RFS) of RCC patients. *GATA3* analysis showed no statistical relationship with survival. In contrast, univariate Cox regression analysis revealed *GATA5* methylation as a strong parameter in the RCC [P<0.001; hazard ratio (HR) = 17.8; 95% (CI) confidence interval, 4.89–65.1] and ccRCC (P<0.001; HR = 13; 95% CI, 3.57–47.4; Table IVA) tissue groups. The Kaplan-Meier analysis with a calculated optimum cut-off of −2.447 for dichotomization showed that higher CGI methylation of *GATA5* is associated with a decreased RFS in patients with ccRCC (Fig. 3E). A pairwise bivariate Cox regression model demonstrated that the *GATA5* CGI methylation status remained a significant and strong parameter in the bivariate models when the status of metastasis, advanced tumor disease, grade, and age were considered as co-variables (Table IVB).

## Discussion

GATA1, GATA2 and GATA3 from the GATA transcription factor family are involved in cellular lineage and hematopoietic development while GATA4, GATA5 and GATA6 are involved in epithelial and endodermal differentiations (13,24). GATA proteins have been suggested to play a crucial role in keeping cells in the undifferentiated state (13). Moreover, previous experiments (10) as well as *in silico* analyses detecting reduced *GATA3* and *GATA5* mRNA expression levels suggested that *GATA3* and *GATA5* are potential targets of epigenetic alteration in RCC. The present study has taken a translational approach to investigate the presence and clinical relevance of CpG island methylation of both genes for RCC.

Tumor cell lines (renal, bladder, prostate and breast cancer) revealed distinct CGI methylation patterns for *GATA3* and *GATA5* methylation but showed no obvious overall correlation between the epi-alterations. Notably, both methylation markers were frequently observed in kidney-derived tumor cell lines and also demonstrated tumor-specific hypermethylation in RCC in concordance with results for our paired group. The present study identified both genes as candidates with a possible relevance for RCC development. Therefore, our data are in line with a recent functional study demonstrating that methylation-dependent silencing of GATA3 expression is correlated with the loss of transforming growth factor-β receptor III and tumorigenesis in ccRCC tissues and cell lines, although its role in disease progression and patient survival remained to be elucidated (25).

Our study revealed that both *GATA3* and *GATA5* showed a highly significant association between CGI methylation and advanced as well as metastatic RCC. Furthermore, *GATA5* CGI methylation exclusively demonstrated a statistical association with grade and lymph node status of the primary tumor. In addition, bivariate Cox regression analysis adjusted for advanced disease, metastatic status, and grade revealed a high and fairly stable HR for *GATA5* methylation in the bivariate statistical survival models overall, identifying this epigenetic mark as a new candidate for independent prognosis of decreased RFS.

Although a great number of hypermethylated loci have been identified in RCC (9), to date, only a subset of CGIs has been functionally or clinically characterized. A recent study found that a large portion of clinically relevant epigenetic alterations identified in RCC also exhibit functional changes in kidney cancer (8). Hence, pre-selection of CGIs based on their statistical association with clinical factors could represent an efficient means of narrowing the pool of candidate epi-alterations affecting the onset or course of RCC. Only a limited number of methylation-based independent candidate prognosticators including *BNC1*, *COL14A1*, *SFRP1*, *SCUBE3*, *GREM1* and *DAL-1/4.1b* (6,8,10,11,26) have thus far been reported. Therefore, our results identify *GATA5* as a new candidate prognosticator gene and suggest its functional relevance in the progression of RCC.

We observed a noticeable difference of approximately two orders of magnitude in the median relative methylation values detected for *GATA3* and *GATA5* CGIs in tumor compared to adjacent normal renal tissues. Considering that histological assessment of control sections ensured a minimum tumor cell content of at least 50% and that identical samples have been measured, a variation in tumor cell content as a possible explanation can be ruled out. Instead, we infer that a different methylation characteristic is present in both CGIs, as detected by qMSP specifically measuring completely methylated sequences. Moreover, as the present study only considered single regions within the analyzed CGIs, we cannot rule out that other methylation marks may exist that exhibit significant associations with clinicopathological parameters, bearing in mind that a recent report has shown such intra-CGI variations (11).

The present study identified *GATA3* and *GATA5* methylation as a common and cancer-specific event in RCC. The association with late-stage disease and for *GATA5* with shortened RFS suggests these targets as biomarkers for biological aggressiveness of RCC and, in case of *GATA5*, as a candidate prognosticator.

## Figures and Tables

**Table I:** Clinicopathological data of patients.

Clinicopathological parameters	*GATA3* (%)	*GATA5* (%)
Cases in total (all RCC)	119 (100)	109 (100)
Histology		
ccRCC	86 (72)	78 (72)
papRCC	24 (20)	22 (20)
Chromophobe/mixed RCC	5 (4)	5 (5)
Not classified	4 (3)	4 (4)
Gender		
Female	42 (35)	37 (34)
Male	77 (65)	72 (66)
Age (years)		
Median	65 (55)	65 (60)
Tumor size		
In diameter (cm)	4.6	4.5
Primary tumor classification		
pT1	11 (9)	11 (10)
pT1a	35 (29)	32 (29)
pT1b	19 (16)	19 (17)
pT2	8 (7)	6 (6)
pT3	5 (4)	4 (4)
pT3a	11 (9)	8 (7)
pT3b/c	25 (21)	24 (22)
pT4	1 (1)	1 (1)
Not known	4 (3)	4 (4)
Lymph node status		
N0	104 (87)	96 (88)
N+	15 (13)	13 (12)
Metastasis classification		
M0	92 (77)	85 (78)
M+	27 (23)	24 (22)
Grade		
Low risk group		
G1	23 (19)	22 (20)
G1–2	16 (13)	14 (13)
G2	60 (50)	57 (52)
High risk group		
G2–3	9 (8)	7 (6)
G3	11 (9)	9 (8)
Localized disease		
pT ≤2, N0, M0 and G1; G1–2	63 (53)	58 (53)
Advanced disease		
pT≥3 and/or N+, M+ or G2–3;G3	55 (46)	50 (46)
Not known	1 (1)	1 (1)
Paired samples		
All RCC	87 (73)	77 (71)
ccRCC	66 (55)	57 (52)

ccRCC, clear cell renal cell carcinoma; papRCC, papillary renal cell carcinoma.

**Table II:** Detailed chromosomal information of *GATA3* and *GATA5*.

	*GATA3*	*GATA5*
Chromosome	10p14	20q13.33
GeneID	2625	140628
CpG Island		
No. of CpG sites	509	247
Base position (bp)	8091375–8098329	61049362–61051897
bp of CpG sites investigated by qMSP	8097735, ~744, ~750, ~796, ~801, ~811, ~831, ~849	61051188, ~210, ~223, ~232, ~236, ~241, ~253, ~255, ~262

Chromosomal information and base position (bp) location of *GATA3* and *GATA5* qMSP relevant CpG sites. Information refers to the UCSC Genome Browser annotation GRCh37/hg 19.

**Table III:** Statistical analyses of *GATA3* and *GATA5* CGI methylation and correlation with clinicopathological parameters in paired t-test and univariate logistic regression analysis.

	*GATA3*		*GATA5*	
Paired t-test	P-value		P-value	
adN/TU				
all RCC	**0.006**		**<0.001**	
ccRCC	**0.001**		**<0.001**	

Univariate logistic regression analysis		OR (95% CI)		OR (95% CI)

ccRCC/papRCC	**0.006**	0.77 (0.63–0.94)	**0.015**	0.80 (0.67–0.96)
Localized/advanced^a^				
all RCC	**0.024**	1.32 (1.04–1.68)	**<0.001**	1.55 (1.29–1.88)
ccRCC	0.277	1.16 (0.89–1.50)	**<0.001**	1.46 (1.19–1.80)
Metastasis: M0/M+				
all RCC	**0.003**	1.59 (1.05–2.43)	**<0.001**	1.65 (1.29–2.11)
ccRCC	0.179	1.38 (0.86–2.20)	**<0.001**	1.64 (1.23–2.17)
Grade: low/high^b^				
all RCC	0.658	1.06 (0.82–1.37)	**0.003**	1.47 (1.14–1.88)
ccRCC	0.542	0.92 (0.68–1.21)	**0.009**	1.54 (1.11–2.14)
Lymph node metastasis: N0/N+				
all RCC	0.187	1.36 (0.86–2.14)	**0.03**	1.32 (1.03–1.68)
ccRCC	0.572	1.17 (0.68–2.01)	0.35	1.15 (0.85–1.56)

adN, adjacent normal tissue; TU, tumor tissue; ccRCC, clear cell renal cell carcinoma; papRCC, papillary renal cell carcinoma; OR, odds ratio; CI 95%, confidence interval.

a Localized tumor is pT ≤2, lymph node (N) and metastasis (M) negative (N0/M0) and grading (G) G1 and G1–2. Advanced tumor is pT ≥3 and/or N+, M+ or G2–3 and G3.

b Low grade tumor (G1 and G1–2). High grade tumor (G2–3 and G3).

**Table IV:** Uni- and bivariate Cox regression model analysis of *GATA5* CGI methylation.

A, Univariate Cox regression analysis of *GATA5* CGI methylation and association with recurrence-free survival in patients with clear cell renal cell carcinoma			

	P-value	HR	95% CI
Methylation	**<0.001**	13.0	3.57–47.4
Status of metastasis (M0/M+)	**0.012**	4.07	1.36–12.2
Localized vs. advanced^a^	0.061	3.44	0.94–12.5
Grade (low/high)^b^	**<0.001**	8.46	2.49–28.7
Age-Median^c^	0.362	0.59	0.19–1.82

B, *GATA5* CGI methylation analysis in a bivariate Cox regression model and its association with recurrence-free survival			

	P-value	HR	95% CI

Methylation	**<0.001**	19.3	4.58–81.6
Status of metastasis (M0/M+)	**0.004**	5.8	1.73–19.4
Methylation	**0.002**	9.55	2.36–38.7
Localized vs. advanced	0.355	1.96	0.47–8.23
Methylation	**0.04**	5.35	1.1–26.1
Grade (low/high)	0.09	3.80	0.80–18.1
Methylation	**<0.001**	29.7	5.72–154
Age-Median^c^	**0.043**	0.23	0.05–0.96

a Localized tumor is pT ≤2, lymph node (N) and metastasis (M) negative. (N0/M0) and grading (G) 1 or 1–2. Advanced tumor is pT ≥3 and/or N+, M+ or G2–3 and G3.

b Low grade tumor (G1 and G1–2). High grade tumor (G2–3 and G3).

c Values dichotomized by the median of parameter.

HR, hazard ratio; CI, 95%, confidence interval. Clinicopathological factors were dichotomized in both regression models.
